# Primary Carcinomas of the Episiotomy Scar Site: A Systematic Literature Review

**DOI:** 10.3390/curroncol32020065

**Published:** 2025-01-26

**Authors:** Andrea Palicelli, Federica Torricelli, Gabriele Tonni, Alessandra Bisagni, Eleonora Zanetti, Magda Zanelli, Venus Damaris Medina-Illueca, Beatrice Melli, Maurizio Zizzo, Andrea Morini, Maria Paola Bonasoni, Giacomo Santandrea, Giuseppe Broggi, Rosario Caltabiano, Francesca Sanguedolce, Nektarios I. Koufopoulos, Ioannis Boutas, Aleksandra Asaturova, Lorenzo Aguzzoli, Vincenzo Dario Mandato

**Affiliations:** 1 Pathology Unit, Azienda USL—IRCCS di Reggio Emilia, 42123 Reggio Emilia, Italy; alessandra.bisagni@ausl.re.it (A.B.); eleonora.zanetti@ausl.re.it (E.Z.); magda.zanelli@ausl.re.it (M.Z.); mariapaola.bonasoni@ausl.re.it (M.P.B.); giacomo.santandrea@ausl.re.it (G.S.); 2 Laboratory of Translational Research, Azienda USL—IRCCS di Reggio Emilia, 42123 Reggio Emilia, Italy; federica.torricelli@ausl.re.it; 3 Department of Obstetrics and Neonatology, Azienda USL—IRCCS di Reggio Emilia, 42123 Reggio Emilia, Italy; gabriele.tonni@ausl.re.it; 4 ISSEMYM Hospital, Toluca 50010, Mexico; vmedinaillueca@gmail.com; 5 Molecular Pathology Unit, Azienda USL—IRCCS di Reggio Emilia, 42123 Reggio Emilia, Italy; beatrice.melli@ausl.re.it; 6 Unit of Obstetrics and Gynecology, Azienda USL—IRCCS di Reggio Emilia, 42123 Reggio Emilia, Italy; lorenzo.aguzzoli@ausl.re.it (L.A.); vincenzodario.mandato@ausl.re.it (V.D.M.); 7 Surgical Oncology Unit, Azienda USL—IRCCS di Reggio Emilia, 42123 Reggio Emilia, Italy; maurizio.zizzo@ausl.re.it (M.Z.); andrea.morini@ausl.re.it (A.M.); 8 Department of Medical and Surgical Sciences and Advanced Technologies “G.F. Ingrassia” Anatomic Pathology, University of Catania, 95123 Catania, Italy; giuseppe.broggi@phd.unict.it (G.B.); rosario.caltabiano@unict.it (R.C.); 9 Pathology Unit, Policlinico Foggia, University of Foggia, 71122 Foggia, Italy; francesca.sanguedolce@unifg.it; 10 Second Department of Pathology, Medical School, National and Kapodistrian University of Athens, Attikon University Hospital, 12462 Athens, Greece; koufonektar@yahoo.com; 11 Breast Unit, Rea Maternity Hospital, P. Faliro, 17564 Athens, Greece; drboutas@gmail.com; 12 1st Pathology Department, National Medical Research Center for Obstetrics, Gynecology and Perinatology Named After Academician V.I. Kulakov of the Ministry of Health of Russia, Bldg. 4, Oparina Street, Moscow 117513, Russia

**Keywords:** episiotomy, carcinoma, endometriosis, clear cell carcinoma, Bartholin, squamous cell carcinoma, endometrioid carcinoma, pregnancy, treatment, imaging

## Abstract

Episiotomy is a perineal incision enlarging the vaginal opening during labor, preventing severe perineal/vaginal/ano-rectal lacerations. We performed a systematic literature review (PRISMA guidelines; Pubmed, Scopus and Web of Science databases) of primary malignant tumors arising from the episiotomy site. Thirteen primary carcinomas were reported, mainly endometriosis-related histotypes (77%) (nine clear cell, CCC; one endometrioid, EC) with only two vulvar invasive squamous cell carcinomas and one adenoid cystic carcinoma of Bartholin’s gland. No sarcomas, melanomas or malignant trophoblastic tumors were described. Endometriosis was associated with tumors or reported in history (62%). Malignant transformation occurred 3 to 27 (mean 16) years after diagnosis of endometriosis. Patients were usually post-/peri-menopausal (eight cases, 61%) (age range: 31–70 years, mean 50). Imaging should exclude distant (0% in our series) or lymph node metastases (three cases, 23%), looking for potential invasion of vagina (five cases, 39%), anus (including sphincter) (four cases, 31%) and/or other deep pelvic soft tissues (five cases, 39%). All patients underwent surgery, except for a CCC-patient (only chemoradiation) subsequently progressing and dying of disease. Adjuvant chemotherapy and/or radiotherapy were administered to five (39%) cases, neoadjuvant therapy to four cases (31%). Globally, three (23%) cases recurred or progressed, and two-thirds (15%) died of disease (1 CCC, 1 EC). Radical surgery with lymph node status evaluation and eventual excision should be performed when possible. Chemotherapy and/or radiotherapy can be considered in an adjuvant and/or neoadjuvant setting (or as only treatment in inoperable patients). However, the role of different treatments should be studied in further larger multicenter series.

## 1. Introduction

Perineal trauma (PT) of any grade after vaginal delivery is a frequent event, involving about 90% of pregnant women, and it may be associated with short- and long-term morbidities (dyspareunia, persistent pain, pelvic floor disorders, depression, negative influence on mother’s ability to care for the newborns, etc.) [1,2,3,4]. The degree of PT-associated morbidity depends on laceration type, repair technique and materials, skill and knowledge of the birth attendant. Risk factors associated with second-degree PT include increased fetal birthweight, operative vaginal birth, prolonged second stage of labor, maternal birth position, and advanced maternal age [1,2,3,4].

Obstetrical anal sphincter injury significantly favor the development of anal incontinence (10% of cases with symptoms within a year from delivery), potentially having medicolegal implications with great impact on healthcare costs (£3.7–9.8 million from 2013 to 2014 in United Kingdom; approximately $83 million between 2007 and 2011 in the United States). Risk factors include induction or augmentation of labor, epidural, increased fetal birthweight, fetal malposition (occiput posterior), operative vaginal birth, midline episiotomy, primiparity and Asian ethnicity. Second-degree perineal tears are more common in primiparous women (incidence: 40%), similarly to obstetrical anal sphincter injury events (6% primiparous vs. 2% multiparous) [1,2,3,4].

Episiotomy is the incision of the perineum to enlarge the vaginal opening during the second stage of labor [1,2,3,4,5,6,7,8,9,10,11,12,13,14,15,16,17,18,19,20,21]. It has a protective role in preventing severe perineal lacerations involving the anal sphincter (3rd-degree) and/or rectal mucosa (4th-degree) [1,2,3,4,5,19,20,21]. For these reasons, this procedure is widely used in Latin America, Europe and United States; however, some studies have identified adverse consequences of episiotomy, such as insufficient prevention of obstetric sphincter ani muscle injuries and hemorrhage [1,2,3,4,5,19,20,21]. Routine episiotomy practice may also increase the risk of major perineal injury according to other studies [1,2,3,4,5,19,20,21,22,23,24,25,26,27,28].

Therefore, the World Health Organization (WHO) and the American College of Obstetricians and Gynecologists (ACOG) have suggested that there are insufficient objective evidence-based criteria to recommend episiotomy as a routine practice, and that clinical judgment remains the best guide for using this procedure; in association with forceps or vacuum delivery, mediolateral episiotomy may have a lower risk of anal sphincter injury than midline episiotomy, but it is associated with an increased likelihood of long-term perineal pain and dyspareunia [29,30,31,32]. Despite these warnings, the prevalence of episiotomy varies widely between countries, from the lowest rate (9.7%) in Sweden to the highest one (100%) in Taiwan according to some studies [33,34].

Pregnancy may also favor the development of new tumors or the malignant transformation of benign conditions such as endometriosis [22,23,24,25,26].

Although rare, benign or malignant tumors have been described to arise from or recur in the episiotomy scar site [6,7,8,9,10,11,12,13,14,15,16,17,18]. In this paper, we have performed a systematic literature review to describe the features and associations of the primary malignant tumors arising from the episiotomy site.

## 2. Materials and Methods

### 2.1. Systematic Review of the Literature

**Figure 1:**
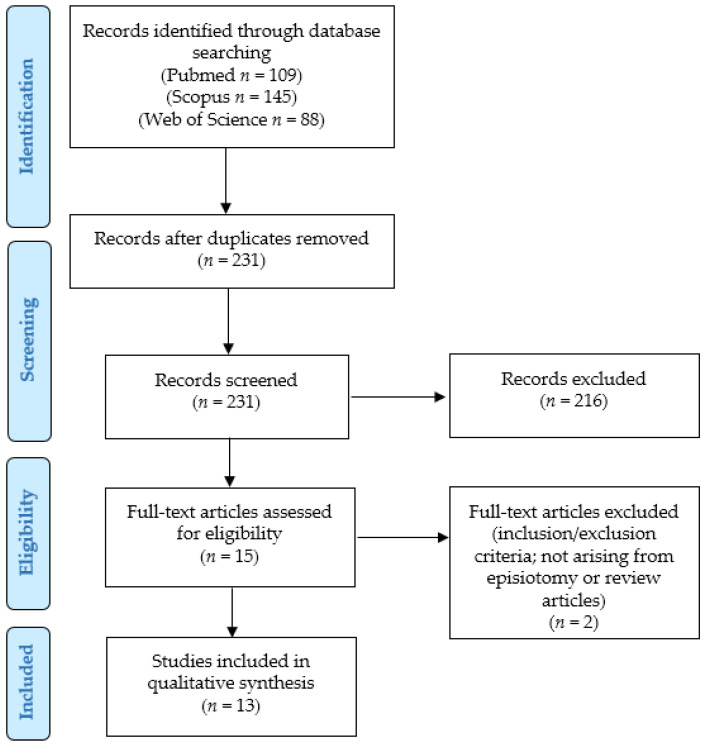
PRISMA flow-chart of our systematic literature review.

To identify primary malignant tumors arising from the episiotomy scar site, we conducted a systematic literature review according to the “Preferred Reporting Items for Systematic Reviews and Meta-Analyses” (PRISMA) guidelines (http://www.prisma-statement.org/; accessed on 2 December 2024) and by using a retrospective observational approach (PICO process) (Figure 1):

- Population: human patients with carcinomas primary arising from episiotomy site;
- Intervention: any;
- Comparison: none;
- Outcomes: clinical outcomes (status at last follow-up, and survival and recurrence rates).

We searched for (carcinoma OR carcinomas OR adenocarcinoma OR adenocarcinomas OR cancer OR sarcoma OR sarcomas OR melanoma OR melanomas OR “gestational trophoblastic” OR choriocarcinoma OR choriocarcinomas OR mole OR molar OR “epithelioid trophoblastic” OR “placental site tumor” OR “placental site tumors” OR “placental-site tumor” OR “placental-site tumors” OR “placental site trophoblastic tumor” OR “placental site trophoblastic tumors” OR “placental-site trophoblastic tumor” OR “placental-site trophoblastic tumors” OR “placental site nodule” OR “placental site nodules” OR “placental-site nodule” OR “placental-site nodules” OR “placental site trophoblastic nodule” OR “placental site trophoblastic nodules” OR “placental-site trophoblastic nodule” OR “placental-site trophoblastic nodules”) AND (episiotomies OR episiotomy) in Pubmed (all fields, 109 results; https://pubmed.ncbi.nlm.nih.gov, accessed on 2 December 2024), Scopus (Title/Abstract/Keywords, 145 results; https://www.scopus.com/home.uri, accessed on 2 December 2024) and Web of Science (Topic/Title, 88 results; www.webofknowledge.com, accessed on 2 December 2024) databases. No limitations were set. The bibliographic research ended on 30 June 2024. We applied the following criteria:

- Eligibility/inclusion criteria: studies describing cases of patients with primary malignant tumors arising in the episiotomy site.
- Exclusion criteria: unclear diagnosis; tumors not primarily arising on episiotomy; results not analyzable (too aggregated or scant data).

After the removal of duplicates, two independent authors read the titles and abstracts of all the retrieved results (*n* = 227). By applying the eligibility/inclusion and exclusion criteria, 15 articles were considered eligible; they were all obtained in full-text format and their reference lists were also screened to search for additional relevant articles. After reading the full-text, 2 papers were excluded as they reported primary vulvar cases not associated with episiotomy [27] or did not describe any additional case [28]; the remaining 13 articles were finally included in our study [6,7,8,9,10,11,12,13,14,15,16,17,18]. The extracted results were checked and confirmed by two other authors.

### 2.2. Statistical Analysis

Data collection was study- and case-related. Age of the patient, clinical symptoms, laboratory and diagnostic investigation such as imaging data, immunohistochemistry, histopathology, treatment, and follow-up information have been evaluated. Categorical variables were analyzed as frequencies and percentages, continuous variables by ranges and mean values.

Statistical analysis was performed using R Foundation for Statistical Computing (R-4.1.3, Vienna, Austria). Distribution of all the continuous variables was tested by the Shapiro test. Associations between clinical and pathological parameters were assessed by the Kruskal-Wallis test for continuous variables and Fisher’s exact test for categorical variables. The overall survival (OS) was computed as the time from the date of surgery to either the date of death or last follow-up, recurrence free survival (RFS) was computed as the time period from the date of surgery to either the date of recurrence or last follow-up. Survival analysis was performed applying by log-rank test. Associations were considered statistically significant for a *p*-value lower than 0.05.

## 3. Results

Globally, we identified 13 primary carcinomas arising from the episiotomy scar site (PriC) [6,7,8,9,10,11,12,13,14,15,16,17,18]. All of the studies reported just one case; the majority of PriCs were equally reported in Europe (*n* = 6; 3 United Kingdom [15,16,17], 1 France [12], 1 Hungary [13], 1 Croatia [14]) and Asia (*n* = 5; 2 Japan [6,9], 2 China [8,10], 1 Korea [11]), while 1 case was described in the United States [18] and Chile [7]. To our review, sarcomas, melanomas or gestational trophoblastic disease (including moles, choriocarcinomas, epithelioid trophoblastic tumors, placental site tumors/nodules) were not reported in association with episiotomy site.

### 3.1. Patient’s Age, Diagnosis and Association with Endometriosis

Globally, PriC patients’ age at presentation ranged from 31 to 70 (mean 50; median 53) years (Table 1) [6,18].

Non-gynecological history details included: 1 previous breast carcinoma (9 years before presentation) (treatment: radical mastectomy + chemotherapy) [8]; 1 hypertension, ischaemic heart disease and psychiatric disease [13]; 1 previous subtotal thyroidectomy for benign node and cholecystectomy [12].

Most of the 13 PriC were histotypes usually related to endometriosis (ERH) (*n* = 10, 77%), including 8 (62%) clear cell carcinomas (CCC) [6,7,8,9,10,11,15,18] and 1 (8%) endometrioid carcinoma (EC) [13]; the remaining case was diagnosed as a serous papillary cystadenocarcinoma but in our opinion, it was likely a CCC or an EC as it was associated with endometriotic foci (unlike serous carcinoma) and the reported immunophenotype did not exclude a CCC/EC (CD10, calretinin, Ki-67) (Table 1) [12].

Indeed, in eight (62%) cases [6,8,10,11,12,13,15,18], endometriosis was associated with the tumor (2 cases, 15%) [6,13], reported in patient’s history at the episiotomy site (1 case, 8%) [18] or both (5 cases, 39%) [10,11,12,15,18]. In two cases, there was evidence of a transitional dysplastic zone between CCC and endometriosis [8,10]. Time from delivery/episiotomy to the histological diagnosis (excision) of endometriosis ranged from 9 months to 12 years (mean 7 years, 83 months) [8,10,11,12,18]. Time from diagnosis of endometriosis to cancer presentation ranged from 3 to 27 years (mean 16 years, 190 months) [8,10,11,12,15,18]. Medical therapy for endometriosis was also administered in 4 cases (Danazol [8,12]; Mifesterone/Medroxy-progesterone acetate injectable suspension, DMPA, followed by mifesterone and Chinese traditional medicine [10]; leuprorelin acetate [11]).

No endometriosis in the three cases revealing endometriosis-independent carcinomas (EIC) (23%), including two vulvar invasive squamous cell carcinomas (VSCC, 15%) [16,17] and one adenoid cystic carcinoma of Bartholin’s gland (ACCBG) [14]. The latter patient had a history of marsupialization of left and right Bartholin’s gland abscesses (nine and seven years before presentation, respectively).

### 3.2. Obstetric History

Most of the patients (61%) were surely postmenopausal (2 cases, 15%; 70 years each) [9,13] or peri-/post-menopausal (6 cases, 46%; decade 50s years) [6,7,8,12,15,17], while five (39%) women were premenopausal (<45 years) (Table 1) [10,11,14,16,18].

Most of ERH patients delivered once (6 G1P1, 60%) [7,8,10,12,15,18] while four (40%) women were multiparous (3 G2P2 [6,9,11], 1 G3P3 [13]); conversely, two-thirds (67%) of EIC were multiparous (1 G2P2 [14], 1 G4P4 [17]) while one woman had at least one previous delivery [16].

Details about episiotomy were available for 8/13 (62%) cases (1 large [7]; 1 left anterior [8]; 2 left mediolateral [9,14]; 1 lateral [10]; 2 right mediolateral [11,18]; 1 right side of introitus [17]). Time from episiotomy to tumor presentation ranged from 3 months to 30 years (mean 20.7 years; median 22 years; available data in nine cases, all being CCCs except 1 ACCBG) [6,8,10,11,12,13,14,15,18]. Three patients had a previous forceps-assisted vaginal delivery [7,10,12].

A PAP smear was performed in one VSCC revealing severe dyskeratosis; recurrent smears with mild abnormalities were found in the previous 12 years [17].

### 3.3. Clinical Presentation

Clinical symptoms at tumor presentation included pain (five cases, 39%) [6,11,12,14,17] in the perineal/episiotomy/tumor area (one prolonged for 10 years [6]; one cyclic [11]; one severe and cyclic pain in seated position, for defecation/miction [12]; one for 18 months [17]; one painful nodule [14]), dyspareunia (two cases, 15%) (one permanent [12], one superficial for two years [16]), abnormal genital bleeding (three cases, 23%) [9,16,18] (one month [9]; one foul-smelling [18]), pruritus (two cases, 15%) [10,15] (one for six months [15]) and vulvar discomfort (one case) [10] (Table 2).

Information about tumor marker levels were available in 5/13 (39%) cases, all being ERHs [6,8,9,12,18]: CA125 serum levels were increased in one-fifth (20%) of cases (79.1 U/mL) [6,8,9,12,18], while CEA (*n* = 4) [6,8,9,18], CA19-9 (*n* = 4) [6,8,9,12], CA15-3 (*n* = 1) [12], SCC (*n* = 3) [6,8,9], AFP (*n* = 2) [8,18], HE4 (*n* = 1) [8], NSE (*n* = 1) [12] and ACE (*n* = 1) [12] levels were always normal.

In all the five cases reporting the clinical suspect, the tumor was misdiagnosed as a benign lesion (*n* = 3; one endometriosis [12], one granuloma [14], one granulation tissue [16]) or a malignant carcinoma of different origin (one anal fistula carcinoma probably as to the involvement of anal sphincter [6]; one Bartholin’s gland cancer, while it was an endometriosis-related CCC [7]).

All of the 13 patients discovered a tumor mass/lesion [6,7,8,9,10,11,12,13,14,15,16,17,18] one month to four years before presentation to clinicians (mean 15 months) (gradual appearing [6]; four years [8]; one month [9]; six months [15]; several months, ulcerated in recent six months [18]; three months [13]; two years [14]). The tumor was described as prominent/exophytic/polypoid [6,9,11,13], nodular [9,14,16,17], hemorrhagic/bleeding [9,11,13], ulcerated/depressed [11,18], with normal/smooth surface [7,9]. Consistency was solid [11,15], hard [12], firm [8,14], tender [17] or soft [8,10], while the tumor color was variably described as tan or red-tan [8], purple [10], bright pink, non-necrotic [13] or dark-reddish [9]. The lesions were well-demarcated/with clear boundaries [9,11], partially mobile [7] or showing infiltrative margins [8].

Globally, the tumor size ranged from 1 to 10 cm (mean 4.6 cm; median 3 cm) (available data in 12/13 cases) [6,7,8,9,10,11,12,13,14,15,17,18]; the mean size was larger for ERH cases (4.4 cm; range 1–10 cm; median 3.5 cm) than for EICs (2.9 cm) but just three cases of the second group gave analyzable data (1.3 cm, 3 cm and 4.5 cm, respectively) [6,7,8,9,10,11,12,13,14,15,17,18].

The tumor site was vulvar [9], perineal [8,10,15,18], vulvar-perineal [7] or vaginal [11,14], and in each case it was related to the episiotomy scar [6,7,8,9,10,11,12,13,14,15,16,17,18]. Six (46%) cases arose from the right side [6,7,11,12,17,18] (including two posterior primary sites) [7,11], while four (31%) tumors were mainly located on the left side [8,9,14,15] and one showed a 1-cm bilateral extension on each side from the midline fourchette [16] (unavailable data in two cases) [10,13].

To better define the tumor features, ultrasound examination (US) was performed in three (23%) cases [8,10,12], magnetic resonance imaging (MRI) in nine (69%) cases [6,7,9,11,12,13,15,18], computed tomography scans (CT) in five (38%) cases [7,8,9,15,18], positron emission tomography (PET) in one (8%) case (uptake 9.13) [9], and PET-CT in three (23%) cases [6,8,12].

Vaginal involvement was reported in five (39%) cases (one vaginal mass, posterior commissure/distal vagina [11]; two cases grew between vagina and anus [6,8]; one upper two-thirds of vagina [13]; one left vaginal introitus [14]), while anal invasion was reported in four (31%) cases (one invasion of anal sphincter/levator ani but involved mucosa [6]; one between the left region of the anus and vagina [8]; one involvement of external anal sphincter and anterolateral anal canal [15]; one right side of anus and distal digestive tract [12]) and five (39%) tumors extended to other deep pelvic soft tissues (one right ischio-anal fossa [7]; one ischio-rectal fossa [18]; two deep pelvis [8,13]; one rectovaginal pouch [15]).

### 3.4. Surgical Treatment and Lymph Node Status

Fine needle aspiration cytology of the tumor was performed in 1 case revealing an adenocarcinoma [6] (Table 3).

Radical/wide surgery was performed in 12 (92%) cases [6,7,8,9,12,13,14,17] (one laparoscopic posterior pelvic exenteration including rectal resection, total mesorectal excision, perineal reconstruction with right gracilis musculocutaneous flap [6]; one with additional V-Y gluteal advancement flap [7]; one radical vulvar excision with skin graft [10]; one radical vaginectomy + wide vulvar excision with partial skin graft + total abdominal hysterectomy, TAH [11]; one also included Hartmann’s procedure, partial vaginectomy and bilateral salpingo-oophorectomy, BSO [15]; one radical vulvectomy [16]) (Table 3).

In three cases, previous gynecological surgery was performed, including TAH + BSO for leiomyomas (*n* = 1) (15 years before) [9], one TAH for adenomyosis and leiomyomas (7 years before) [15] and one prolapse surgery (laparoscopic suspension procedure + posterior vaginal colpoperineoplasty) (4 years before) [12].

In seven (54%) cases, the surgical margins were free of tumor [9,10,11,12,14,16,17] (unavailable data in the remaining cases).

Enlarged inguinal lymph nodes were found in four (31%) cases (two left [8,9], one bilateral [18], one unclear [6]); in two-quarters (50%) of cases, this enlargement was clinically evident [9,18] while it seemed identified only by MRI or CT in the two other patients which also revealed uptake of fluorodeoxyglucose (FDG) on PET/CT and metastases in the lymphadenectomy specimen [6,8]. Internal iliac lymph node enlargement was additionally identified by MRI in one case [6]. In 6/13 (46%) cases, lymph nodes were not enlarged [7,10,11,12,15,17] while data were unavailable in the remaining three cases [13,14,16].

FNAC was performed on inguinal lymph nodes in two (15%) cases [8,11]; one was suggestive for an epithelial neoplasm (subsequent lymphadenectomy was positive for metastasis) [8], while the second case was negative for neoplasia, but subsequent lymphadenectomy revealed a metastasis [11].

An inguinal lymph node was biopsied in four (31%) cases [6,9,12,18]: in all the cases, the biopsy result agreed with the following lymphadenectomy specimen. In particular, two cases revealed a metastatic carcinoma [6,18], and two biopsies were negative for neoplasia (one found endometriosis) [9,12].

Globally, inguinal lymphadenectomy (LND) was performed in seven (54%) cases [6,8,9,10,11,16,17] (three bilateral [6,8,16], one left LND + right inguinal lymph node biopsy [9], one right [11], one unclear side [10], one superficial and deep ipsilateral inguinofemoral [17]) while additional pelvic LND was carried out in two (15%) cases (one bilateral [6], one unclear site [11]).

### 3.5. Stage at Presentation

The pTNM/AJCC category was difficult to apply, as the pT category was not provided in any case, the endometriosis-related histotypes are unusual in the vulvar site, the descriptions were sometimes difficult to interpret, and it was sometimes unclear which could represent the primary site (vulva? vagina?).

Globally, three cases were pN+ (1 bilateral inguinal and right internal iliac [6], one bilateral superficial inguinal, one lymph node per site [8], one right inguinal lymph node biopsy [18]) and 4 (31%) cases were N0 [9,11,12,17], while data were unavailable in two cases (Table 3) [10,16]. In four (31%) cases, lymphovascular space invasion was not identified [9,11,14,16], while data were not available in the other cases. One tumor showed perineural invasion [14], but this information was not highlighted in the remaining cases.

Distant metastases at presentation were not reported in any case [6,7,8,9,10,11,12,13,14,15,16,17,18].

### 3.6. Neoadjuvant and Adjuvant Treatment

Four (31%) patients underwent neoadjuvant treatment with gonadotropin releasing hormones (Gn-RH) (one case) [12], chemotherapy (paclitaxel + cisplatin, one cycle) (one case) [10], chemotherapy (5-fluorouracil with leucovorin and oxaliplatin, mFOLFOX6) + panitumumab (seven courses) (one case, initially misdiagnosed as an anal fistula adenocarcinoma, achieving partial response) [6] or radiotherapy (RT) (19 fractions, then discontinued for severe skin reaction; partial response) [15].

While primary surgery was performed in 12 (92%) cases [6,7,8,9,12,13,14,17], one patient did not undergo surgery and only chemoradiation was administered achieving partial response; later, she progressed and died of disease [18].

Adjuvant chemotherapy was administered to three (23%) patients (one paclitaxel + carboplatin, four cycles [6]; paclitaxel + cisplatin, one cycle [10]; one carboplatin, weekly [12]) while adjuvant RT was performed in one of these cases (perineal area and inguinal LNs, 45 Gy in four weeks + interstitial application of iridium) [12] and in two other cases (unclear data; one month duration in one case) [8,13] (total: three cases, 23%).

### 3.7. Follow-Up

Follow-up data were available for all 13 cases, ranging from 5 to 30 (mean 11.7, median 11) months (Table 3) [6,7,8,9,10,11,12,13,14,15,16,17,18]. Globally, 3 (23%) cases recurred or progressed [13,14,18], while 10 (77%) cases did not recur [6,7,8,9,10,11,12,15,16,17].

Eight ERH cases and three EIC cases showed no evidence of disease from five to fifteen (mean 7.8) months [6,7,8,9,10,11,12,15] and eleven to thirteen (mean 12) months after diagnosis [14,16,17], respectively. One of these cases showed local recurrence after six months, but the patient was surgically treated with hemivulvectomy + left LND (pN0) followed by RT achieving complete response and being free of disease thirteen months after diagnosis [14].

Only two (15%) cases progressed (one CCC: locally and in the lungs, after twelve months [18]; one EC: unclear sites and timing [13]) and died of disease thirty months (CCC) [18] and twelve months (EC) [13] after diagnosis, respectively.

### 3.8. Immunohistochemical and Molecular Analysis

Immunohistochemistry was tested on six (43%) cases, including four CCC [6,7,8,9], the serous carcinoma (probable CCC) [12] and the ACCBG [14]. The following markers were positive in all the few tested ERH cases: PAX-8 (*n* = 3) [6,7,9], HNF1-β (*n* = 3) [6,8,9], CA125 (*n* = 1) [9], napsin (*n* = 1) [7], pan-cytokeratin and AMACR (*n* = 1) [8]. Hormone receptors are typically negative in CCCs, but can sometimes be positive: estrogen receptor (ER) resulted positive in both the tested cases [6,9], but the intensity and extension of staining was not completely clear. Conversely, progesterone receptor (PR) was negative in the only tested case [8], as well as GATA-3 [9], SALL-4 [9], PTEN [9], PAX-2 [9], AFP [9] and calretinin [12], while ARID1A was retained in one case [9] and another tumor has a CK7+/CK20− phenotype [6]. As expected, the two tested CCCs resulted in p53 wild-type [8,9], while an unclear positivity for p16 was reported in one of the two cases [8,9]. No clear data about Ki67 were available. CD10 was tested in three cases [6,9,12] but it was not always clear if positivity was evident in the tumor or in the endometriotic stroma.

Two CCC were examined by molecular analysis [6,9]. One case revealed no *KRAS* mutation [6], while no pathogenic/oncogenic mutations in 50 cancer-related genes (such as *BRAF*, *EGFR*, *ERBB2*, *FBXW7*, *GNAS*, *HRAS*, *KIT*, *KRAS*, *NRAS*, *PIK3CA*, *PTEN*, *APC*, *CTNNB1*, and *TP53*) were detected in the second case by targeted sequencing (Ion Ampliseq Cancer Hotspot panel version 2) [9].

The ACCBG case showed the following immunophenotype: EMA + (few luminal cells), pan-cytokeratin + (few luminal cells), smooth muscle actin + (basaloid peripheral cells), S100 (basaloid peripheral cells) ER −, PR − [14]. Moreover, flow cytometry was performed revealing a DNA diploid tumor with low S-phase fraction of 3.43% [14].

### 3.9. Statistical Analysis

All the following relevant variables were analyzed for association with histology (each histotype; ERH vs. EIC), OS and RFS: mean age, primiparous vs. multiparous, left/right side of episiotomy, endometriosis associated with tumor and/or present in history or absent, time from diagnosis of endometriosis to tumor presentation, time from episiotomy to tumor presentation, involvement of deep pelvis tissues, anus or vagina, enlarged lymph nodes, estimated pT stage, pN stage, radical surgical excision, lymphadenectomy, neoadjuvant treatment (Cht or RT), neoadjuvant Cht, neoadjuvant RT, adjuvant treatment (Cht or RT), adjuvant Cht, adjuvant RT, Cht (neoadjuvant or adjuvant), RT (neoadjuvant or adjuvant). Moreover, associations of histotype (ERH vs. EIC) with OS and RFS were investigated. However, no statistical significance was identified (*p* < 0.05) for all the statistical analyses, probably because of the low number of reported cases.

## 4. Discussion

In our review, we identified only 13 primary carcinomas arising from the episiotomy scar site [6,7,8,9,10,11,12,13,14,15,16,17,18] while no other malignant non-carcinomatous histotypes (such as sarcomas, melanomas or trophoblastic tumors, which rarely arise from extrauterine gynecologic organs) [35,36,37,38,39,40,41,42,43,44] resulted from our search to be associated with the episiotomy site.

The three endometriosis-independent histotypes arose one decade earlier if compared to ERHs (43 vs. 53 years) and may have been related to HPV infection (VSCC, 15%) [16,17] or chronic inflammation/stimulation of Bartholin’s glands (history of marsupialization for abscesses) (one ACCBG) [14]; representing exceptional events, they may be incidental findings not significantly associated with episiotomy.

Chronic inflammation and autoimmune diseases are well-known risk factors for tumor development as they facilitate tumor progression and treatment resistance, while acute inflammation stimulates dendritic cell maturation and antigen presentation, leading to anti-tumor immune responses [45,46].

Similarly, some reports suggested that HPV infection (not confirmed in our cases) may be associated with poor healing of the episiotomy repair, thus favoring chronic inflammation [47,48]. Moreover, episiotomy and Bartholin’s gland marsupialization may favor local inflammation and the arising of fistulae which may represent a chronic stimulus and/or cover the presence of an underlying neoplasm; however, it is difficult to speculate on the relevance of these features in our rarely reported cases [49,50].

Conversely, most (*n* = 10/13, 77%) of the described cases seemed to arise from a background of endometriosis [6,7,8,9,10,11,12,13,15,18], which was identified in association with the tumor or in history in the majority of cases (*n* = 8, 62%) [6,8,10,11,12,13,15,18]. These ERHs were typically CCCs, with just one EC [6,7,8,9,10,11,12,13,15,18]. Molecular and immunohistochemical data were too scant to classify these tumors according to the molecular classification proposed for the similar endometrial carcinoma histotypes [51,52,53,54,55,56].

Endometriosis is a chronic, estrogen-dependent, inflammatory disease defined by the implantation of ectopic endometrial glandular and stromal cells outside the uterine cavity [57,58,59,60,61,62,63,64,65,66,67,68]. Endometriosis affects about 5% to 15% of women, prevailing in the reproductive age (25–38%) and presenting with severe dysmenorrhea, dyspareunia, infertility and/or chronic pelvic pain; however, it can also continue to cause morbidities in post-menopausal women [49,50,51,52,53,54,55,56,57,58,59,60]. In our series, pain either in the vulvar-perineal/episiotomy/tumor (five cases, 39%) [6,11,12,14,17] or in the form of dyspareunia (two cases, 15%) [12,16] was the most common symptom, followed by abnormal genital bleeding (three cases, 23%) [9,16,18], pruritus (two cases, 15%) [10,15] and vulvar discomfort (one case) [10].

Endometriosis usually occurs in pelvic sites (ovaries, Fallopian tubes, peritoneum, uterine serosa, round ligament, uterosacral ligament, pouch of Douglas, rectovaginal septum, etc.) but it is not unusual to find endometriotic foci in upper abdomen (peritoneum, gastrointestinal or genitourinary tracts, etc.), while extra-abdominal sites may also be rarely involved (vulva, lymph nodes, thorax/lung/pleura, diaphragm, nervous system, mucocutaneous tissues, etc.) [57,58,59,60,61,62,63,64,65,66,67,68]. Globally, extrapelvic endometriosis seems to occur in about 20% of women with endometriosis [57,58].

In particular, vulvar-perineal endometriosis affects less than 1% of patients and it can be due to traumatic and/or iatrogenic seeding of endometrial tissue during vaginal deliveries, episiotomy (incidence: 0.03–0.15%) or other surgical or obstetrical procedures (cesarean section, myomectomy, hysterectomy) [23,57,58,59,60,61,62,63,64,65,66,67,68,69,70,71,72]. In our series, time from delivery/episiotomy to the histological diagnosis of endometriosis ranged from 9 months to 12 years (mean 7 years, 83 months) [8,10,11,12,18]. The vulvo-perineal damaged mucosa can favor the implant of endometriotic foci. Viable endometrial tissue may also circulate into the peritoneal fluid. Rare cases maybe be spontaneous and unrelated to these risk factors potentially supporting the hypothesis of a lymphatic or hematogenous dissemination theory and/or the possibility of a direct extension of endometrial mucosa from the pelvis to the vulvo-perineal region through the round ligament/Nuck canal. Moreover, the cellular immunity theory suggests that a deficiency in immunity cells may favor the proliferation of ectopic endometrial tissue [57,58,59,60,61,62,63,64,65,66,67,68].

Luckily, malignant transformation of endometriosis is uncommon (1% of cases); this event is more frequent in the ovaries (80% of the endometriosis-associated malignancies) while it is extremely rare in extra-gonadal endometriosis [68,69,70,71,72,73,74,75,76,77,78,79,80,81].

Hyperestrogenism and long-lasting unopposed estrogen therapy (22 months to 17 years) may favor the malignant transformation of endometriosis, especially in case of estrogen-responsive ECs [69,73,74,75,76,77,78]. In contrast, the high physiological concentration of progesterone typical of pregnancy can antagonize the proliferation of estrogen-dependent tumor cells, a well-known mechanism that allows fertility to be preserved in endometrial cancer patients [82]. Thus, multiparous women have a lower risk of developing cancer; in our series, most of ERH patients delivered once (six cases, 60%) [7,8,10,12,15,18] while two-thirds (67%) of EIC were multiparous [14,17].

However, CCC commonly arises from endometriosis (4.5% of extragonadal ERHs) and typically show estrogen receptors (ERs) downregulation with lack the immunohistochemical ER expression; moreover, mesonephric-like adenocarcinomas, a rare new entity which also downregulate ER and PR, can also exceptionally arise from endometriosis [69,73,74,75,76,77,78,79,80,81,82,83,84]. In particular, in some series, CCCs represent the most frequent histotype [85]. Indeed, almost all of our ERHs were CCCs and most of the patients were surely postmenopausal (two cases, 15%) [9,13] or peri-/post-menopausal (six cases, 46%) [6,7,8,12,15,17], while five (39%) women were premenopausal (<45 years) [10,11,14,16,18].

The etiopathogenesis of these histotypes associated with endometriosis but not with ER expression is still under evaluation. Repeated bleeding of endometriosis during the menstrual cycle or retrograde menstruations cause changes in the microenvironment and carry highly pro-oxidant factors into the ovarian endometrioma or peritoneal cavity. In particular, this cyclic or persistent inflammation causes heme and redox active iron-mediated oxidative stress which modifies DNA, proteins and lipids, favoring DNA damage or loss of heterozygosity, as well as aberrant methylation of CpG islands or other epigenetic changes of a number of key regulatory genes. The loss of ER expression seems to be a result of the hypermethylation of the ER-α promoter. Other epigenetic events including histones deacetylation and methylation, and the effects of PPAR-γ and ubiquitin protein ligases, are involved in the complex mechanism regulating promoter transcription and driving stable gene expression modifications. The switch from a normal-stress-response phenotype to a stress-resistant phenotype may induce gene mutations (such as *ARID1A*, *PIK3CA*, *PPP2R1A*, *KRAS*, etc.) causing progression to CCC. Immune cell dysfunction and inflammation may play a role as well [86,87,88,89,90,91,92].

Malignant transformation of endometriosis seems to more frequently occur some years after the diagnosis of endometriosis. A systematic review of abdominal wall endometriosis found that time between first surgery to malignant transformation ranged from 4 to 41 (mean 19.9) years [85], while in our series time from diagnosis of endometriosis to cancer presentation ranged from 3 to 27 (mean 16) years [8,10,11,12,15,18].

Especially in patients with a history endometriosis, the appearance of a new nodule at the site of episiotomy or an increase in size of a pre-existing small lesion could represent a benign (inflammatory or endometriotic) lesion; indeed, three cases of our series presented with this clinical suspect [12,14,16]. However, an accurate gynecological investigation should be performed in these women with vulvo-vaginal-perineal cyclic pain or nodules, to prevent potential complications such as malignant transformation. Not all endometriotic lesions need to be resected but follow-up and biopsy of selected cases may be considered; a complete resection (achieving free resection margins) of endometriotic nodules increasing in size, long-standing, with persistent symptoms and/or resistant to medical therapy, can be recommended to reduce the risk of persistence and recurrence, as well as the rare possibility of malignant transformation [81,93].

Some studies reported that cancers associated with endometriosis have a better prognosis than those not associated [80,94]; only two (15%) of our cases progressed and died but larger series are needed as, unfortunately, our sample size was too small.

All primary vulvar carcinomas may potentially involve the episiotomy site, and the rarity of some episiotomy-associated tumors could not exclude incidental findings as abovementioned [14,16,17,47,48,49,50,95]. One CCC of our series was clinically suspected to represent a Bartholin’s gland cancer [7].

Moreover, cancers arising from other organs (gastro-intestinal, gynecological, genitourinary tracts, breast, etc.) may rarely metastasize (synchronously or metachronously) to unusual sites, including the vulva, sometimes mimicking a primary Paget’s disease or revealing the subtle primary origin after careful clinical and imaging investigations [95,96,97,98,99,100,101,102,103,104,105,106,107,108]; conversely, vulvar cancers may also metastasize to uncommon sites, potentially mimicking another primary tumor [109,110,111]. One of our cases was indeed misdiagnosed as an anal fistula carcinoma, probably due to the involvement of anal sphincter [6].

We feel that a point of strength of our literature review is the multidisciplinary detailed description of the state of the art about this infrequently analyzed topic through a systematic search in multiple databases according to the PRISMA guidelines, which include an evidence-based minimum set of items for reporting and are useful for the critical evaluation of the submitted manuscripts; indeed, systematic literature reviews and meta-analyses are increasingly relevant to keep clinicians up-to-date, also representing a starting point for developing clinical practice guidelines or further studies/trials [112,113,114,115,116,117,118,119,120,121,122,123].

The rarity of episiotomy-associated tumors has not allowed us to get significant results from statistical analysis, representing the main limit of drawing clear conclusions and clinical indications from our results. As just 13 cases were previously described (typically as case reports), the risk of malignant transformation for patients who underwent episiotomy seems low, but publication selection bias due to the choices of authors and policies of scientific journals may also have underestimated the incidence of this event.

The provided clinic-pathologic data were sometimes scant or questionable, representing another limit of our analysis. For example, the pT stage category was not provided in any case and the proper pTNM/AJCC stage was difficult to apply in most of the cases. In addition, some histotypes lack detailed immunohistochemical and molecular data, including a case with a maybe questionable histologic diagnosis [12]. Sometimes, it was also unclear if the tumor was centered on the vulva, vagina or perineum. For all these reasons, the statistical analysis was not applicable to evaluate our data with reliable significance.

Geographic variations in the frequency of performing episiotomy, as in the incidence of tumors and their risk factors, may represent another bias for our analysis and may affect the generalizability of the findings. Conditions other than episiotomy identified in our series may represent the real risk factors or co-factors for malignant transformation, as a synergistic effect cannot be excluded. However, they also may just represent incidental findings.

Future larger multicenter series with case centralization for histopathological review and/or analysis may be helpful to identify the real incidence of PriCs and to verify if this procedure increases the risk of cancer development [124,125,126].

Imaging (CT, PET and MRI) can exclude distant (0% in our series) or lymph node metastases (three cases, 23%) [6,8,18] and can define the potential tumor invasion of vagina (five cases, 39%) [6,8,11,13,14], anus (including sphincter) (four cases, 31%) [6,8,12,15] and/or other deep pelvic soft tissues (five cases, 39%) [7,8,13,15,18].

When achievable after complete staging, radical local surgical excision with lymph node status evaluation and eventual excision should be performed in order to avoid recurrence and progression; adjuvant and/or neoadjuvant treatment can be administered to improve the survival and disease-free recurrence rates, but no clear surgical or oncologic guidelines could be defined due to the few cases described. Globally, three (23%) cases recurred or progressed [13,14,18] and two of them (15%) died of disease [13,18]. One relapsing ACCBG-patient was surgically treated with hemivulvectomy + left LND (pN0) followed by RT achieving complete response and being free of disease 13 months after diagnosis [14]. Surgery was performed in all cases except for a CCC-patient who just underwent chemoradiation, achieving partial response and subsequently showing lung progression after one year and dying of disease [18]. The last recurring patient with EC died of disease after one year [13]. Globally, three cases were pN+ [6,8,18]; two cases treated with lymphadenectomy showed no evidence of disease after 15 months [6,8] while the remaining case was the non-surgically treated progressing and dying patient [18]. Adjuvant treatment (chemotherapy and/or RT) was administered to five (39%) cases [6,8,10,12,13], while neoadjuvant therapy was occasionally administered (four cases, 31%) [6,10,12,15].

## 5. Conclusions

Primary carcinomas arising from episiotomy were rarely reported in literature. Most of the cases were CCCs in post-/peri-menopausal patients that arose from endometriotic foci. Malignant transformation is a rare and not early event (mean 16 years after diagnosis of endometriosis). The diagnosis may be late when the disease is already in an advanced stage. At follow-up, a careful gynecological examination should be performed, and complete resection of long-standing endometriosis may be considered.

For tumors arising from episiotomy, complete imaging staging and exclusion of a vulvo-perineal metastasis from neoplasms arising from other primary sites are mandatory. Radical surgery with lymph node status evaluation and eventual excision should be performed when possible. Chemotherapy and/or radiotherapy can be considered in an adjuvant and/or neoadjuvant setting (or as only treatment in inoperable patients). However, the impact of surgery, radiotherapy and chemotherapy should be studied further in larger series with a multicenter and multidisciplinary study approach.

## Figures and Tables

**Table 1:** Primary carcinomas arising from the episiotomy scar site.

Case	Age	Diagnosis	History	Endometriosis	Episiotomy Type	Time from Episiotomy to Tumor Presentation (mo)
1. Kasahara et al., 2021 [6]	53	CCC (*)	2 vaginal deliveries with E (26 and 23 years before); prolonged pain (E, 10 years)	TA	NR	312
2. Barrena-Medel et al., 2020 [7]	54	CCC (*)	Forceps-assisted vaginal delivery	no	Large	NR
3. Xu et al., 2020: case 1 [8]	54	CCC (*)	Breast carcinoma (9 years before presentation; radical mastectomy + chemotherapy); vaginal delivery + E (30 years before). Endometriosis at E: (1) 3 years post-E, 27 years before presentation: excision; (2) few months later: excision; (3) persistence: Danazol (1 year)	TA (°), H	Left/anterior	360
4. Kojima et al., 2019 [9]	70	CCC (*)	G2P2; TAH + BSO (15 years before for leiomyomas)	no (§)	Left mediolateral	NR
5. Han et al., 2016 [10]	36	CCC (*)	Forceps delivery (20 years before): slow postoperative perineal wound recovery. Frequent vaginitis (bad health habits; lack of treatment). Cyclic E/perineal pain/swelling (several months after delivery; relieved by Mifesterone and DMPA). Vulvar discomfort and pruritus (frequent scratching of vulva/E for many years). Excision of E-endometriosis (9 years before diagnosis; 11 years after previous delivery), then DMPA (12 mo), Mifesterone (6 mo), and Chinese traditional medicine.	TA (°), H	Lateral	240
6. Kwon et al., 2008 [11]	42	CCC (*)	2 previous normal spontaneous vaginal deliveries (last, 15 years before). Previous endometriosis (right posterior commissure/E; 3-cm vaginal cystic mass; pruritus, intermittent postcoital bleeding; cyclic severe pain; treatment: excision + leuprorelin acetate for 6 mo, 36 mo before presentation).	TA, H	Right mediolateral	216
7. Todd et al., 2000 [15]	54	CCC (*)	Excision of endometriosis in E (25 years before); TAH (adenomyosis, leiomyomas) (7 years before)	TA, H	NR	>300
8. Hitti et al., 1990: case 3 [18]	43	CCC (*)	P1001; normal spontaneous delivery (15 years before); Previous endometriosis (E, 7 years before; 8 years after delivery)	H	Right mediolateral	180
9. Chene et al., 2007 [12]	50 (p)	SC (CCC?) (*)	Complicated delivery (forceps extraction; 3200 g female, 30 years before); perineal/E/pelvic endometriosis (24 years): follow-up + excision of painful E- nodule (6 years after delivery), vaporization of pelvic endometriosis, danazol; subtotal thyroidectomy (benign node); cholecystectomy; prolapse surgery (laparoscopic suspension procedure + posterior vaginal colpoperineoplasty) (4 years before)	TA, H	NR	360
10. Nagy P., 2003 [13]	70	EndC (*)	G3P3 (between 43 and 48 years of age); hypertension, ischaemic heart disease; psychiatric patient	TA	NR	>264
11. Krasević et al., 2003 [14]	31	ACCBG	G2P2; marsupialization of left and right Bartholin’s gland abscesses (9 and 7 years before)	no	Medio-lateral (left)	3
12. Olah et al., 1995 [16]	44	VSCC (G2) + VIN	at least G1P1; superficial dyspareunia (2 years); menorrhagia	no	NR	NR
13. Van Dam et al., 1992: case 1 [17]	53	Early invasive VSCC	G4P4; PAP test: severe dyskeratosis; recurrent smears with mild abnormalities (12 years)	no	Introitus (right side)	NR

(p): perimenopause; (*): histotype associable with endometriosis. (°): evidence of transitional dysplastic zone between CCC and the endometriotic focus; (§): severe fibrosis; no intraepithelial lesion. ACCBG: Adenoid cystic carcinoma of Bartholin’s gland; BSO: bilateral salpingo-oophorectomy; CCC: clear cell carcinoma; DMPA: Medroxy-progesterone acetate injectable suspension; E: episiotomy; EndC: endometrioid carcinomas; H: history of endometriosis; mo: months; NR: not reported; SC: Serous papillary cystadenocarcinoma; TA: associated with tumor; TAH: total abdominal hysterectomy; VIN: vulvar intraepithelial neoplasia; VSCC: vulvar squamous cell carcinoma.

**Table 2:** Primary carcinomas arising from the episiotomy scar site: clinical presentation, size and tumor site.

Case	Presentation	Size (cm)	Site
1 [6]	Prominent mass (GE); prolonged pain at E (10 years); increased CA125 (79.1 U/mL); normal CEA (1.2 ng/mL), CA19-9 (26.1 U/mL) and SCC (1.1 ng/mL)	4	E (right, between vagina and anus); invasion of anal sphincter/levator ani muscle
2 [7]	Partially mobile mass (normal skin, no inflammation)	7	Vulvoperineal, posterior (right ischio-anal fossa)
3 [8]	Mass (GE, 4 years; soft then firm, infiltrative margins, tan or red-tan); normal CA125, AFP, CA-19-9, CEA, HE4 and SCCA	6	Left perineal (related to anterior E) extending to deep pelvis between anus and vagina
4 [9]	Bleeding nodule (exophytic sessile; dark-reddish; clear boundary and smooth surface; FDG standardize uptake: 9.13); abnormal genital bleeding (1 mo); normal CEA, CA19-9, SCC, and CA125	1.8	Vulva, left vestibulum/labium minora
5 [10]	Mass (soft, purple scar), vulvar discomfort and pruritus	10	Perineum, E-apex
6 [11]	Mass (solid, well-demarcated, hemorrhagic, polypoid and ulcerated/depressed), cyclic perineal pain	3	Vagina (right/distal; posterior commissure; E)
7 [15]	Mass (solid, painless, pruritic perineal) (GE, 6 mo)	3	Left perianal/E, rectovaginal pouch; involved external anal sphincter and anterolateral anal canal
8 [18]	Mass (several mo; ulcerated in recent 6 mo; unresponsive to Danazol); foul-smelling bloody discharge; normal OC-125, CEA, AFP	1	Perineum/inner right buttock; extension to ischio-rectal fossa
9 [12]	Mass (hard); severe perineal pain (cyclic pain in seated position, for defecation, miction, permanent dyspareunia); normal CA125, CA15-3, CA19-9, ACE, and NSE.	3.5	E, right anus and distal digestive tract
10 [13]	Mass (exophytic, bright pink, non-necrotic, hemorrhagic); vaginal discharge (3 mo)	3	E, extension to upper 2/3 of vagina and pelvic organs
11 [14]	Nodule (firm; 3 mo after delivery, painful 2 years later	1.3	E (left vaginal introitus)
12 [16]	Nodule	NR	E (fourchette, bilaterally extending for 1 cm)
13 [17]	Nodule (tender); pain (E, 18 mo)	4.5	E (right side of introitus) (maybe also perineum and vagina)

E: episiotomy; GE: gradual enlargement of the tumor; mo: months; NR: not reported.

**Table 3:** Primary carcinomas arising from the episiotomy scar site: lymph node status, surgical treatment, tumor recurrence and follow-up information.

Case	Enlarged LNs	LN Status	Treatment	RM	Rec	FU (mo)
1 [6]	Right In and internal iliac LN (MRI; PET-CT uptake)	pN+ (bilateral In and right internal iliac)	FNAC (tumor) + biopsy of right inguinal LN (G3 ADK); neoadjuvant ChT (mFOLFOX6 + panitumumab, 7 courses) (#) + laparoscopic posterior pelvic exenteration (rectal resection, total mesorectal excision, perineal reconstruction with right gracilis musculocutaneous flap) + bilateral lateral pelvic and In LND + ChT (paclitaxel + carboplatin, 4 cycles)	NR	no	NED, 15
2 [7]	no (In)	N0	Radical excision + V-Y gluteal advancement flap	NR	no	NED
3 [8]	Left In (CT: 3 cm; PET/CT: increased uptake of 18FDG)	pN+ (superficial In: 1 right, 1 left)	FNAC (In LN: positive) + radical excision + bilateral In LND + RT (1 mo)	NR	no	NED, 15
4 [9]	Left In (clinical, CT)	pN0 LVI-	Incisional biopsy; radical local excision + left In LND + right In LN biopsy	Negative	no	NED, 5
5 [10]	no	Nx	Excisional biopsy; neo-ChT (paclitaxel + cisplatin, 1 cycle); radical vulvar excision (skin graft) + In LND + ChT (paclitaxel + cisplatin, 1 cycle)	Negative	no	NED, 6
6 [11]	no (MRI)	pN0 LVI-	Incisional biopsy; radical vaginectomy + WE (partial skin graft) + TAH + pelvic LND + right In LND	Negative	no	NED, 10
7 [15]	NR	Nx	biopsy; RT (19 fractions, then discontinued for severe skin reaction; partial response) + radical excision (including Hartmann’s procedure, partial vaginectomy, BSO)	NR	no	NED, 6
8 [18]	bilateral In (clinical)	N+	incisional biopsy of tumor mass and enlarged right In LN; ChT + RT (partial response)	Surgery not performed	PD (12 mo; local, lung)	DOD, 30
9 [12]	no (In)	pN0	GnRH; LN biopsy; complete resection + RT (perineum In LNs; 45 Gy in 4 weeks) + ChT (carboplatin weekly) + BT (interstitial application of iridium)	Negative	no	NED, 6
10 [13]	NR	Nx	Excision + RT	NR	PD	DOD, 12
11 [14]	NR	pN0 LVI- (°)	WE Recurrence: hemivulvectomy + left LND + RT	Negative	local (6 mo)	NED, 13
12 [16]	NR	Nx, LVI-	Excision + radical vulvectomy + bilateral In LND	Excision: positive (VSCC); vulvectomy: negative	no	NED, 12
13 [17]	no (In) (clinical and imaging)	pN0 (°)	CB (normal) + excision (E); WE (E); superficial and deep ipsilateral inguinofemoral LND	Negative	no	NED, 11

(°): other information about stage included: prominent perineural invasion [14], tumor size 4.5 mm, depth of invasion 1.8 mm [17]. (#): misdiagnosed as anal fistula ADK [6]. ADK: adenocarcinoma; BSO: bilateral salpingo-oophorectomy; BT: brachytherapy; CB: cone biopsy; ChT: chemotherapy; CT: computed tomography scan; E: episiotomy scar lesion; FNAC: fine needle aspiration cytology; In: inguinal; TAH: total abdominal hysterectomy; LN: lymph node; LND: lymph node dissection; LVI: lymphovascular invasion; mFOLFOX6: 5-fluorouracil with leucovorin and oxaliplatin; mo: months; MRI: magnetic resonance imaging; NED: no evidence of disease; NR: not reported; PET: positron emission tomography; Rec: recurrence; RM: resection margins; RT: radiotherapy; VSCC: vulvar squamous cell carcinoma; WE: wide local excision.
